# Presentation and challenges in management of COVID-19 in sickle cell disease: case report

**DOI:** 10.11604/pamj.2023.46.25.32057

**Published:** 2023-09-15

**Authors:** Sheliza Parvez Thaver, Saliha Shafik Dawood, Nahida Zahir Walli

**Affiliations:** 1 Aga Khan Hospital Dar-es-Salaam, Dar-es-Salaam, Tanzania

**Keywords:** Sickle cell disease, COVID-19, paediatrics, Tanzania, case report

## Abstract

Sickle cell has predominantly been a disease in the sub-Saharan Africa. There is scanty data in Africa and none in Tanzania regarding patients with sickle cell disease infected with COVID-19 especially in the paediatric group. This is concerning because a large population of children living with sickle cell disease are found in this part of the world where scarcity of resources also prevail. This is the first paediatric case of sickle cell disease and COVID-19 reported in Tanzania; highlighting the presentation and challenges faced in management. It is also the first case in literature describing secondary infection in a child with COVID-19 and underlying sickle cell disease. Our patient was a female child of African origin with underlying sickle cell disease who had recurrent admissions. She presented with cough, fever, chest and abdominal pain and was managed for acute chest syndrome and vaso-occlusive crisis. During the second admission, she developed respiratory distress, infection with COVID-19 was confirmed and managed accordingly. However, she was re-admitted due to bilateral arm pain and physical findings were suggestive of secondary respiratory bacterial infection. She was then started on a different treatment plan. Despite challenges faced in the management of the patient, the outcome was favourable. It is important to recognize the presentation of COVID-19 in children with sickle cell disease and challenges faced in management so that the epidemiologic characteristics, spectrum of the disease and its outcomes can be understood better in the context of sub-Saharan Africa.

## Introduction

The highest burden of sickle cell disease (SCD) is in Africa where up to 75% of the 300,000 global births of SCD per year occur [1]. A study conducted in Northern Tanzania reported birth prevalence of sickle cell disease to be 3.9%. This is the highest prevalence ever reported in Tanzania and among the highest recorded in sub-Saharan Africa [2]. Although sickle cell disease patients are considered high risk group for severe illness with COVID-19, available evidence in literature suggests that majority of children with sickle cell disease infected with COVID-19 tend to have better clinical evolution. However, most of the studies that have established this largely originate from outside sub-Saharan Africa. There is dearth of information on COVID-19 and sickle cell disease in the paediatric group especially in sub-Saharan Africa. The child reported in our case presented with recurrent hospitalization that posed a serious challenge in management of the patient. First challenge was encountered due to overlap in presentation of COVID-19 and acute chest syndrome. Second challenge was faced when managing secondary infection. However, the outcome of our patient was favourable. This is the first paediatric case of sickle cell disease with COVID-19 reported at a private institution in Tanzania since the beginning of the pandemic. According to our knowledge, it is also the first case in literature describing a secondary infection in a child with COVID-19 and sickle cell disease.

## Patient and observation

**Figure 1:**
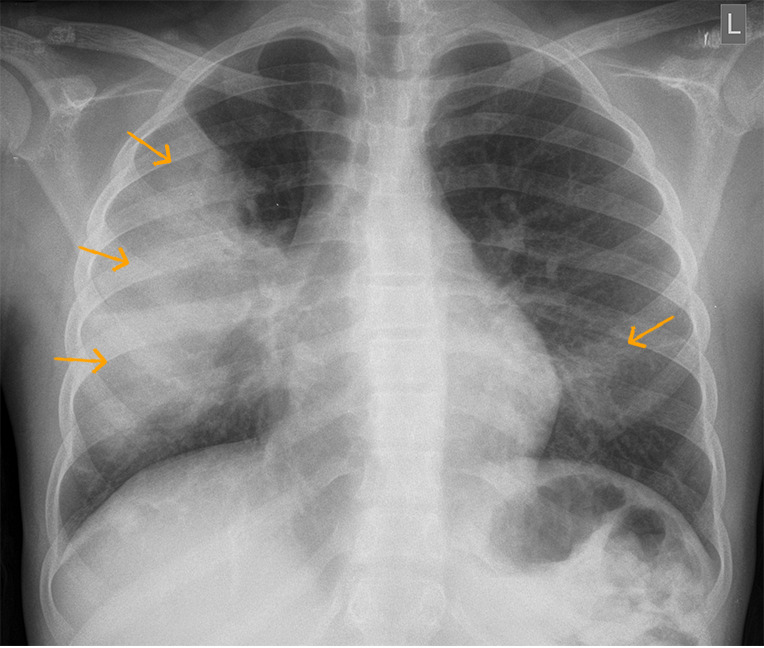
chest X-ray-lung fields showing a large right mid zone consolidation (arrows right) with no significant loss of volume; few left lower zone infiltrates are noted as well (arrow left)

A thirteen-year-old, Tanzanian female child known to have sickle cell disease was seen at the outpatient clinic in late July 2021 due to cough and high grade fever and was prescribed intravenous ceftriaxone. The child´s Hb electrophoresis record indicated homozygous sickle cell disease (HbSS). She did not have history of recurrent sickle cell crisis, hence she had stopped using hydroxyurea for more than six years. Her baseline hemoglobin has been 7.5g/dL. However, symptoms persisted with onset of mild chest and abdominal pain and was admitted at Aga Khan Hospital in the Paediatric Ward in Early August 2021. The child had no signs of respiratory distress, saturations in room air were 98% but had reduced air entry on the right side of the chest with crepitation. Chest x-ray showed a large right mid zone lung consolidation with few left lower zone infiltrates noted (Figure 1). A diagnosis of vaso-occlusive crisis and acute chest syndrome was made and patient managed with Intravenous (IV) Piperacillin and Tazobactam 100mg/kg every six hours, oral azithromycin 10mg/kg once daily, IV fluids, IV paracetamol 15mg/kg and oral ibuprofen 10mg/kg around the clock for pain control. During the course of stay in the hospital, fever, cough and pain subsided and patient discharged after seven days.

**Clinical findings:** two days later, she was re-admitted due to severe chest and abdominal pain with tachypnea and desaturation in room air. Diffuse crepitation was heard on both sides of the chest.

**Timeline of events:** (Table 1).

**Table 1:** timeline of events

Date	Summary of hospitalizations and follow-up visits	Diagnostic testing	Intervention
**2021/07/30**	Initial outpatient visit-Presented with persistent high-grade fever and cough. Diagnosis: Bacterial infection	WBC: 21.24*109/L, Neutrophil Abs count-16.15*109/L, HB-8.7g/dL	Started on Ceftriaxone
**2021/08/01**	Hospitalized for above symptoms + mild abdominal & chest pain, reduced air entry on right of chest with crepitation. Diagnosis: Acute Chest Syndrome & Vaso-Occlusive Crisis	WBC : 24*109/L, Neutrophil Abs count-19.08*109/L, HB-7.4g/dL, CRP-198mg/L, Blood culture-No bacterial growth, Chest X-ray- large right mid zone lung consolidation with few left lower zone infiltrates	Started on Piperacillin & Tazobactam, Azithromycin, IV fluids, Paracetamol and Ibuprofen
**2021/08/08**	Doing well, fever and pain subsided. Patient discharged	NONE	Discharged with Co-amoxyclav, Hydroxyurea and Analgesics
**2021/08/10**	Re-admitted due to severe chest & abdominal pain, tachypnea and desaturation. Diagnosis: COVID-19 infection	WBC : 39*109/L, Neutrophil Abs count-36.30*109/L, HB-7.4g/dL, CRP-202mg/L, Blood culture-No bacterial growth, CT-Scan- left lower lobe consolidation accompanied by peripheral sub-pleural patchy ground glass opacities with right peri-bronchial ground glass, traction bronchiectasis, D-dimer-5.43, Ferritin-436.37, LDH-666.60IU/L, Swab for COVID-19 PCR-Positive	Kept on oxygen via nasal prongs 3L/min, Started on Meropenem, Clarithromycin, Therapeutic dose of Enoxaparin, Dexamethasone, Vitamin supplements, Paracetamol, Ibuprofen and Morphine
**2021/08/14**	Respiratory distress and pain subsided. Patient discharged	CRP-136.25mg/L	Discharged with Meropenem, Clarithromycin, Oral Dexamethasone, Rivaroxaban, Vitamin Supplements & Analgesics
**2021/08/20**	Follow up visit-Child doing well. Chest clear on auscultation	CRP-12.28mg/L	Continued with Meropenem
**2021/08/22**	Re-admitted due to bilateral arm pain, chest exam-new crepitation heard on right side of chest. Diagnosis: Secondary respiratory infection	CRP-108mg/L, Procalcitonin-0.25ng/L, Blood culture-No bacterial growth	Continued with Meropenem, Clindamycin added, Paracetamol, Ibuprofen and Morphine
**2021/08/28**	Stable and discharged	CRP-70mg/L	Discharged with Clindamycin and Analgesics
**2021/09/02**	Follow up visit-Child doing well	NONE	NONE

WBC-White Cell Count, HB-Hemoglobin, CRP-C-Reactive Protein, LDH-Lactate Dehydrogenase, PCR-Polymerised Chain Reaction

**Figure 2:**
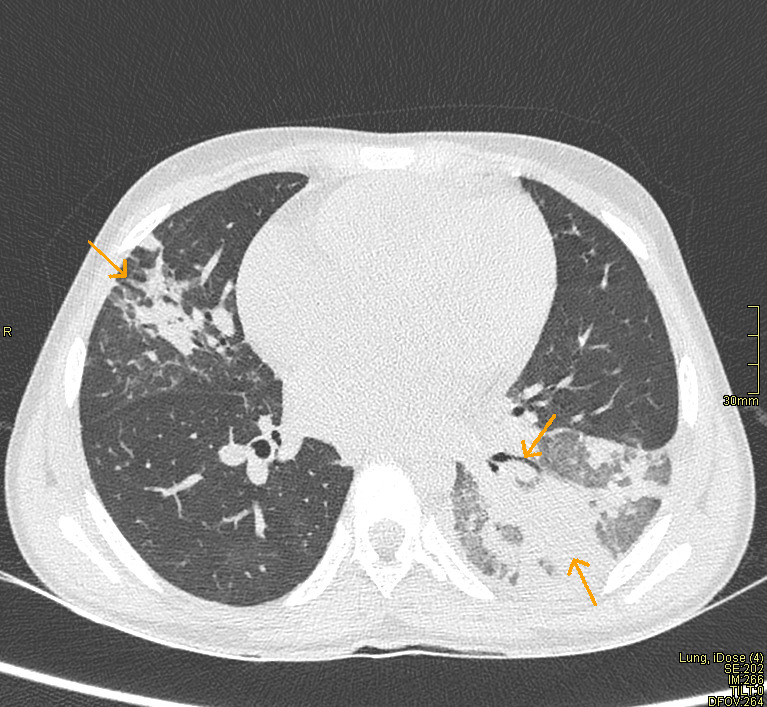
CT scan-axial image demonstrating left lower lobe consolidation (arrow on left-below) with air bronchogram (arrow on left-above) accompanied by peripheral sub-pleural patchy ground glass opacities (arrow right)

**Diagnostic assessment:** computed Tomography (CT) scan was done due to suspected pulmonary embolism which was ruled out but revealed left lower lobe consolidation with air bronchogram accompanied by peripheral sub-pleural patchy ground glass opacities (Figure 2). Laboratory studies showed white cell count of 39*10^9^/L with neutrophil absolute count of 39*10^9^/L, hemoglobin of 7.4g/dL and CRP of 202mg/L. In view of the above findings, a high index of suspicion for COVID-19 was made and oropharyngeal swab for COVID-19 Polymerase Chain Reaction (PCR) was obtained which confirmed the diagnosis. Additional supportive findings included a D-dimer of 5.43, ferritin of 436.37 and lactate dehydrogenase of 666.60IU/L.

**Therapeutic intervention:** she was kept on oxygen via nasal prongs 3L/min, IV meropenem 20mg/kg every eight hours, oral clarithromycin 7.5mg/kg every twelve hours, subcutaneous enoxaparin 1mg/kg every twelve hours, oral dexamethasone 0.15mg/kg once daily, vitamin supplements, IV fluids, IV paracetamol 15mg/kg, oral ibuprofen 10mg/kg and IV morphine 0.1mg/kg around the clock for pain control. On the third day, oxygen was weaned off, chest and abdominal pain gradually subsided. She was discharged on the fourth day on meropenem to complete a total of 14 days, clarithromycin, oral dexamethasone to complete total of 10 days, rivaroxaban, vitamin supplements and analgesics. Parental education was given on hand hygiene, adherence to face masks and isolation of the child at home.

**Follow-up and outcomes:** her follow up visits revealed that she was responding well to the treatment and the chest findings were normal on auscultation with CRP level going down up to 12.28mg/L. Treatment adherence and tolerability were assessed by close follow up. However, she was re-admitted after seven days from discharge of the second admission due to bilateral arm pain. Physical examination revealed new onset of crepitation heard on the right basal lung field without respiratory symptoms. CRP was elevated to 108mg/L. Procalcitonin level was checked and was found to be 0.25ng/L. Blood cultures were negative again. IV clindamycin 30mg/kg every 8 hours was initiated in suspicion of secondary respiratory bacterial infection due to Methicillin Resistant Staphylococcus aureus (MRSA), IV fluids, IV paracetamol 15mg/kg, oral ibuprofen 10mg/kg and IV morphine 0.1mg/kg for pain control. She was discharged after one week once pain subsided and continued with oral clindamycin to complete total of 14 days. Her follow up visits showed that she had been recovering well after the initiation of clindamycin.

**Patient perspective:** the child was anxious due to recurrent illness but with her parents’ support and counselling provided by the medical team, she felt emotionally better. Her parents were aware of the challenges faced in management and were supportive and satisfied with the management offered to their child.

**Informed consent:** written informed consent was obtained from the patient´s next of kin for publication of this case report and any accompanying images.

## Discussion

A study done in Senegal described COVID-19 infection to be severe in children with sickle cell disease whereby one patient died [3]. However, majority of studies available in literature have revealed that children with COVID-19 and sickle cell disease recovered and showed a better clinical evolution [4-6]. Despite recurrent hospitalization of our patient, the outcome was favourable. Data on COVID-19 pneumonia and bacterial co-infection/secondary infection in children are sparse. There are no African reports to date [7]. A meta-analysis done in China showed bacterial co-infection in 3.5% of patients and bacterial secondary infection in 14.3% of patients infected with COVID-19 [8]. However, this study was described in children and adults having COVID-19 infection, including those with other comorbidities but not sickle cell disease.

A study done in adults with COVID-19 infection revealed most infections to be secondary-microbiologically confirmed (70.6%). 84.9% of culture positive respiratory samples represented secondary infection with gram negative organisms being the predominant pathogens followed by *Staphylococcus aureus* [9]. Another study showed prevalence of bacterial superinfection from respiratory samples in patients hospitalized for COVID-19 to be 49.6% with *Klebsiella (pneumonia* and *oxytoca*) and *Staphylococcus aureus* being the most frequent pathogens [10]. Again, these studies were conducted in the adult population with other comorbidities, some mentioned as immunosuppression but not a mention of sickle cell disease. Given the increased prevalence of respiratory bacterial superinfection noted among adults with COVID-19 and increased risk of infections among individuals with sickle cell disease due to splenic dysfunction, further research is required among the paediatric group diagnosed with COVID-19 infection and having sickle cell disease in order to explore the burden of secondary infections among them. During the third admission of our patient, the CRP level was elevated again but because of its non-specificity, procalcitonin level was obtained which was 0.25ng/L. This value was not very suggestive of a bacterial infection. But, due to the onset of new crepitation heard on auscultation and the possibility of fever being masked due to the usage of analgesics which also serve as anti-pyretics, a secondary respiratory bacterial infection was suspected. In view of the microbes responsible for this infection, clindamycin was initiated presumptively for MRSA coverage and the patient responded well to treatment. This was yet another challenge in the management of the patient. The strength of the study was that we were able to confirm COVID-19 infection in our patient and management given accordingly.

One limitation in our case report was that, the child was started on empiric treatment with clindamycin to cover for MRSA. Culture of sputum or deep respiratory secretion was not done to confirm the diagnosis. This was because of lack of skilled personnel to perform broncho-alveolar lavage and partly because of risk of spread of COVID-19 by aerosol generating procedures. It should be noted that the testing capacity for COVID-19 and its coverage is low in Tanzania and other sub-Saharan African countries. In addition, sophisticated radio-imaging and laboratory investigations as well as drugs for management of COVID-19 are not widely available in these countries. This raises concern about how many missed cases there may be which are not diagnosed or managed effectively. Due to overlap in symptoms of acute chest syndrome and COVID-19, patients with sickle cell disease presenting with respiratory symptoms should ideally be referred to and evaluated at a tertiary hospital. However, due to constraints in health care system and finances in sub-Saharan Africa, those with severe respiratory symptoms should be immediately referred. Those with mild-moderate respiratory symptoms should be isolated and managed according to protocols. Such protocols should be created keeping in mind the capacity of the health care systems and the affordability of the citizens. As it is said, prevention is better than cure. Thus, children with sickle cell disease should be educated about COVID-19 and its implications, advised to wear face mask outdoors, frequent sanitising of hands and have their regular medications stocked at home.

## Conclusion

Evaluation and management of children with sickle cell disease infected with COVID-19 can be challenging. Due to relatively high rates of sickle cell disease in sub-Saharan Africa and similar clinical presentations between acute chest syndrome and COVID-19, all children with sickle cell disease presenting with respiratory symptoms should ideally be tested for COVID-19. Such cases that are seen in this part of the continent should be published in literature so that the management would depend on the unique epidemiologic characteristics, spectrum of the disease, availability of resources and health care financing in sub-Saharan Africa. Also further research needs to be done on COVID-19 and secondary infection among children with sickle cell disease in order to better understand the risks, microbiological profile, management and outcomes.
